# Nested PCR-Based Rapid Detection of Phytoplasma Leaf Wilt Disease of Coconut in Sri Lanka and Systemic Movement of the Pathogen

**DOI:** 10.3390/pathogens12020294

**Published:** 2023-02-10

**Authors:** Prasad R. De Silva, Chandrika N. Perera, Brian W. Bahder, Renuka N. Attanayake

**Affiliations:** 1 Crop Protection Division, Coconut Research Institute, Lunuwila 61150, Sri Lanka; 2 Department of Agricultural Biology, Faculty of Agriculture, University of Peradeniya, Peradeniya 20400, Sri Lanka; 3 Department of Entomology and Nematology, FLREC-University of Florida, Davie, FL 33314-7719, USA; 4 Department of Plant and Molecular Biology, University of Kelaniya, Kelaniya 11600, Sri Lanka

**Keywords:** phytoplasma, coconut, leaf wilt disease, nested PCR, 16S rRNA

## Abstract

Phytoplasmas are associated with many plant diseases. In palms, lethal bronzing disease, Texas Phoenix palm decline, and coconut lethal yellowing decline are some of them. In Sri Lanka, coconut leaf wilt decline has been reported in the Weligama area of the Southern province, and the disease is called Weligama coconut leaf wilt disease (WCLWD). Unlike other phytoplasma diseases of palms, WCLWD shows slow disease progress. Pathogen detection entirely relies on nested polymerase chain reaction (PCR). However, inconsistencies in pathogen detection have been experienced, i.e., symptomatic plants often produce negative results. The objectives of this study were to reconsider the choice of primers and to determine the best sampling tissue types for consistent detection of the pathogen. Among the six universal primer combinations tested, P1/Tint nested with fU5/rU3 produced consistent results. BLASTn searches of the sequences showed 99–100% similarity to sugarcane white leaf disease (SWL) or grassy shoot (SGS) disease-causing phytoplasma. The optimized nested PCR protocol was successful, with the minimum success rating of 88% and 100% specificity. Midribs of milky white bud leaf samples were the best tissue type for rapid detection. Systemic movement of the pathogen and a tentative latent period were also reported. The findings are helpful in the early detection of the disease.

## 1. Introduction

Since the first identification of phytoplasma in 1967 [1], nearly 300 diseases caused by phytoplasmas have been recorded in more than 1000 plant species [2,3]. Phytoplasma damage is significant due to its worldwide distribution on economically important plants such as sugarcane and various palm species. Coconut lethal yellowing (LY) is one such well-studied phytoplasma disease in most coconut-growing countries around the world [4,5]. Based on the 16S rDNA sequences, the pathogen is grouped as 16SrIV, and so far, about 20 major groups have been reported on various hosts [4]. A high degree of divergence was reported among different coconut phytoplasmas making pathogen detection challenging [6]. A range of symptoms, including the premature drop of most or all fruits, brown or black water-soaked appearance at the calyx end of the nut, inflorescence necrosis, foliar discoloration, yellowing and withering of the crown, hanging down withered fronds making a skirt appearance around the trunk several weeks before falling, and leaf flaccidity, is associated with affected palms [4]. These symptoms may vary slightly with the palm species and the variety affected and the environmental conditions [4].

First identified in 2006 in the Weligama area of Sri Lanka’s Southern province [7,8,9], Weligama coconut leaf wilt disease (WCLWD) is one such phytoplasma disease. Currently, it is a quarantine disease in Sri Lanka due to its devastating nature. Infected plants lose the angular shape of the leaflets, resulting in a flattened appearance, which is termed flaccidity. Flaccid leaflets tend to bend downward, giving a ribcage-like appearance. This is the initial symptom of the disease. The most noticeable secondary symptom is the intense and unusual yellowing of lower fronds and a drastic reduction of yield. However, by then, the plant is severely affected and ultimately prone to other infections like leaf rot that result in the death of the palm. Complete removal of infected palms has been the only recommendation; accordingly, more than 340,000 coconut palms have been cut and removed in the southern region of the country [8]. Early, accurate, and precise detection is a preliminary requirement in disease management. However, early detection is not easy for an average person, and molecular-based detection is required. Detection is achieved by polymerase chain reaction (PCR) of the 16S rRNA region and sequence comparison. The pathogen was first detected in 2008 using nested PCR with universal primers: R16mF2/R16mR1 [10] nested with fU5/rU3 [11], R16F2n/R16R2 nested with fU5/rU3 [12], and P1/P7 [13] nested with Chrfor/rU3 [14], as well as Pc399/P1694 [15]. In 2012, Perera et al. identified the pathogen using 16S rRNA sequence information and recognized that WCLWD belonged to the 16SrXI group [7]. However, the success rate of pathogen detection was only 54%, emphasizing the importance of revisiting the detection method. Since this first report, no population-scale systematic study has been conducted. Recently, population-scale pathogen detection was attempted to determine the current status of the disease in the country, and inconsistent results were observed. In certain instances, obviously symptomatic plants recorded negative PCR results and vice versa. This could be due to the lower specificity of the primers, uneven titer and the erratic distribution of the pathogen in the plant, variability in primer-binding sites, or the association of other fastidious prokaryotes with the symptomatic samples. Even though immunological detection methods [16] and a qPCR method [17] have been developed, they are not cost-effective and feasible for large-scale studies. Therefore, further improvements to the PCR-based method are required. Therefore, this study was mainly aimed at the selection of suitable primer pairs and sampling tissue types for convenient and reliable detection of the pathogen.

Once phytoplasmas are transmitted via sap-sucking insects [18], a considerable period would lapse before the appearance of visual symptoms in coconut palms, and it is referred to as the latent period. The latent period varies depending on the taxonomic group of infecting phytoplasma, virulence, age and species of the palm, and the climatic conditions. An incubation period of 114–191 days has been reported for lethal yellowing (LY) disease in Jamaica [19], whereas a six-month to three-year incubation period has been reported for Kerala root wilt (KRW) in India [20]. However, no information is available on the incubation period of WCLWD in Sri Lanka.

To address these questions, firstly, it is essential to develop a consistent pathogen detection strategy with high reproducibility. Trunk-boring samples have been widely used in detecting coconut/palm lethal yellowing disease in many countries [21]. However, the best sampling tissue type for consistent pathogen detection of WCLWD is not known.

The key objectives of this study were to re-evaluate the suitability of different primer combinations for consistent and convenient pathogen detection, determine the best tissue type for PCR, infer pathogen movement and systemic nature of the infection, tentatively identify the incubation period in the field, and determine whether other fastidious prokaryotes are associated with WCLWD using universal primers. The overall goal of the study was to make pathogen detection convenient and consistent.

## 2. Materials and Methods

### 2.1. Pathogen Detection Using Nested PCR

Commercial coconut plantations with a history of WCLWD were selected from the Kotawila area in the Matara district in the Southern province of Sri Lanka. Sampling for pathogen detection was performed in February 2021. Twenty-five milky white bud leaf samples from WCLWD-symptomatic palms and 10 samples from asymptomatic palms from a WCLWD-free area were used in the experiment. The selected coconut palms were at least 10 m apart from each other. The tissues sampled were kept in polyethylene zip lock bags separately, transported to the laboratory, and stored in a refrigerator for DNA extraction. In total, 35 tissue samples were used for assay development. The leaves were thoroughly washed with distilled water and with 70% ethanol and cut into small pieces. Ekel (midrib) was separated from milky white bud leaves and ground into a fine powder using a mortar and pestle with liquid nitrogen and 0.2 g of the fine power were used for DNA extraction. DNA extraction was completed using a modified CTAB method [22]. The quality and the quantity of extracted DNA were measured using a Nanodrop (Nanodrop 2000, USA) and stored at −20 °C until further use.

For pathogen detection, nested PCR was conducted using various combinations of primer pairs to amplify the 16S rRNA region as shown in Table 1. The PCR master mixture was prepared with 1× Go Taq Flexi buffer (Promega, Madison, WI, USA), MgCl_2_ (0.6 to 1.5 mM), 0.5 µM of each primer, dNTP (150 to 200 µM), template DNA (1–4 µL), and GoTaq DNA polymerase (1–1.25 U) (Promega, Madison, WI, USA). The concentration of each component was adjusted as per the original publications shown in Table 1. Sterilized distilled water was used as the negative control and the DNA extracted from the leaf tissues with symptoms of sugarcane white leaf disease or sugarcane grassy shoot disease was used as the positive control [7,23]. PCR reactions were carried out in a 96-well thermal cycler (Takara Bio Inc., Otsu, Japan) using the following conditions: 35 cycles with denaturation at 94 °C for 30 s, annealing for 45 s at 56–60 °C (Table 1), extension at 72 °C for 1 min, and final extension at 72 °C for 10 min, followed by storage at 4 °C.

Altogether, six primer pair combinations were tested for each sample. PCR products were subjected to electrophoresis on 1% agarose gel under 90 V for 1 h (Labnet, Taiwan, China) and visualized with a UV transilluminator (Cell Biosciences, UK). Clear DNA bands resulting from PCR amplification were subjected to bidirectional Sanger sequencing at Macrogen Inc., Seoul, Republic of Korea, and the results were compared with the published records available in the NCBI database using BLASTn searches. For phylogenetic analysis, the sequences of the current study along with the previously published and several unpublished sequences of phytoplasmas of palms, sugarcane, and one sequence from mulberry were used. As an outgroup for the analysis, the 16S rRNA sequence of *Acholeplasma palmae* (L33734.1) was used. Table 2 shows the sequence accession numbers, references, and host plants used in the analysis.

Sequence editing and assembly were carried out using BioEdit 7.2.5 software [31]. The sequences were aligned and the maximum likelihood unrooted tree was constructed using Molecular Evolutionary Genetics Analysis (MEGA) X software [32,33]. The evolutionary history was inferred by using the maximum likelihood method and the Tamura–Nei model. The stability of the inferred subclades was evaluated with 1000 bootstrap replications. The initial tree(s) for the heuristic search were obtained automatically by applying the neighbor-joining and BioNJ algorithms to a matrix of pairwise distances estimated using the maximum composite likelihood (MCL) approach and then selecting the topology with the superior log likelihood value.

### 2.2. Assessment of Tissue Types for Pathogen Screening

Twelve WCLWD-symptomatic palms and 12 asymptomatic palms were selected for this purpose. In addition, six healthy palms were also selected from a WCLWD-free area, Lunuwila in Puttalam district in Sri Lanka, as controls. The total sample size consisted of 30 palms. From each palm, four different tissue types (bud leaves, young inflorescence (two months before opening), stem borings, and roots) were taken separately, making a total of 120 samples. Leaf tissues were cleaned and subjected to DNA extraction following the same procedure as described in the previous experiment. Rachilla of young inflorescence and roots were surface-sterilized thoroughly by washing with running tap water followed by 70% ethanol for 2 min. Tissues were cut into small pieces (2–5 mm). Stem borings were taken from each palm separately by drilling the trunk using a handheld power drill. A drill bit of 13 mm diameter was used and stem borings were taken from 2–3 inches inside the trunk. The drill bit was surface-sterilized by dipping it in 70% ethanol between samplings. Stem borings were kept in zip lock bags separately and transported to the laboratory. All the samples were ground into a fine powder using a mortar and pestle with liquid nitrogen separately and 0.2 g of the fine powder was used for DNA extraction. Finally, 120 tissue samples (bud leaf tissues, inflorescence tissues, stem borings, and root tissues; 30 each) were subjected to PCR with the most reliable primer pair identified above. PCR conditions were the same as above. In brief, the first PCR cycle was carried out with the P1/Tint [12] primers in a 30 µL final reaction mixture. Later, nested PCR was carried out using 1–2 µL of the PCR product as the template DNA and with the best nested primer pair. The PCR products were subjected to 1% agarose gel electrophoresis and visualized as described above.

### 2.3. Screening for Other Fastidious Prokaryotes

For all the above-mentioned DNA samples extracted from all of the tissue types (*n* = 120), PCR was conducted separately using other fastidious prokaryote-specific universal primers targeting five genera reported to infect annual plants: *Spiroplasma, Xylella*, *Clavibacter, Phlomobacter*, and *Liberibacter.* PCR reaction conditions were the same as described in the relevant literature cited in Table 3 and sterilized distilled water was used as the negative control.

### 2.4. Pathogen Movement within Palms and the Latent Period

Temporal sampling was performed to determine the pathogen movement and whether an incubation period is associated with the disease prior to visual symptoms expression. For this experiment, six WCLWD-asymptomatic coconut palms were selected from the WCLWD-affected area and two sampling rounds were completed in a one-year period. All the experimental palms were photographed for the observation of visual symptoms development. Tissues from bud leaves, young inflorescence, stem borings, and roots were sampled during each sampling round. A total of 48 samples were used for the study. After extracting genomic DNA, the samples were subjected to nested PCR as described above.

## 3. Results

**Figure 1:**
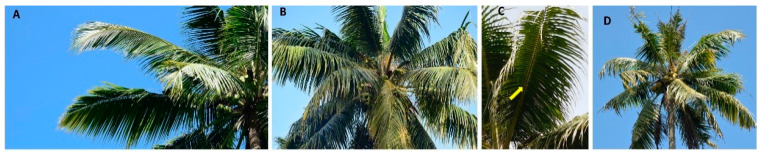
Symptoms of a WCLWD-affected coconut palm: (**A**) a healthy coconut palm, (**B**) an infected coconut palm showing early symptoms, (**C**) the ribcage-like structure of leaves (arrowhead), (**D**) a WCLWD-infected coconut palm superimposed with leaf rot.

Loss of the angular shape of the leaflets resulting in a flattened appearance or flaccidity, which is a typical symptom of the disease, was observed in the selected symptomatic plants. Flaccid leaflets have given a ribcage-like appearance of an animal. The palms weakened by WCLWD are often prone to other leaf rot diseases, and such palms were not included in the sampling (Figure 1).

### 3.1. Pathogen Detection Using Nested PCR

Out of the six primer combinations tested, positive results were observed only for the P1/Tint and fU5/rU3 universal phytoplasma-specific primer pairs in nested PCR (Table 1). Out of the 25 WCLWD-symptomatic bud leaf samples, 22 showed positive results, recording a success rate of 88%. All the 10 healthy bud leaf samples tested from the disease-free area showed negative results, giving 0% false positive detection. The amplified products were in the expected range of 800–900 bp.

**Figure 2:**
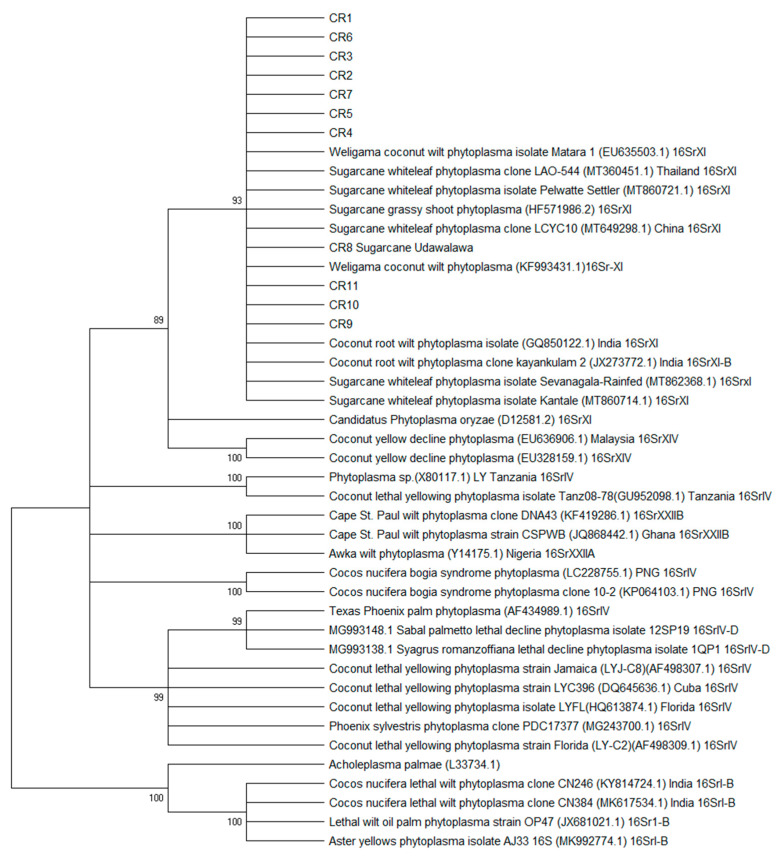
Phylogenetic tree inferred using the maximum likelihood approach of 16S sequences of phytoplasma. A condensed unrooted tree showing 85% cutoff values is shown. Bootstrap support above 85% is shown on each branch.

The phylogenetic placement of the samples of WCLWD with other phytoplasmas was also analyzed in this study (Figure 2). A condensed maximum likelihood tree with branches showing more than 80% bootstrap support is shown below. All the Sri Lankan samples were similar to each other and clustered together with sugarcane white leaf disease-causing phytoplasma group 16SrXI.

The tree with the highest log likelihood (−4350.35) is shown. The percentage of trees in which the associated taxa clustered together is shown next to the branches. The tree is drawn to scale, with branch lengths measured in the number of substitutions per site. This analysis involved 44 nucleotide sequences and a total of 1255 positions in the final dataset. Evolutionary analyses were conducted in MEGA X [32,33].

All the WCLWD sequences were similar to each other and showed 99–100% similarity to the sugarcane white leaf disease (WLD) or sugarcane grassy shoot disease (GSD) phytoplasma samples deposited in the GenBank with accession numbers MT862368.1, MT86721.1, MT860714.1, HF571986.2, MT360451.1, and MT649298.1. Since not many phytoplasma sequences are available in published records in GenBank, only a few published sequences were used in the phylogenetic analysis. All the Sri Lankan samples as well as the sugarcane samples clustered together with 93% bootstrap support in the 16SrXI group. All the other groups were clustered separately with high bootstrap support.

### 3.2. Selection of a Tissue Type for Pathogen Detection

PCR-based detection of phytoplasma in each tissue type varied as shown in Table 4. The highest detection rate was observed in bud leaf samples (75%) followed by the inflorescence samples and roots and the stem borings had the lowest detection rate. Therefore, ekels of milky white bud leaves were found to be the most reliable type of plant tissue for consistent pathogen detection. The apparently healthy tissues collected from disease-prevalent areas also showed the presence of the pathogen in PCR tests, indicating that although the plants were asymptomatic, the pathogen might have been undergoing a latent period in such palms. However, no pathogen was detected in any sample type collected from the disease-free area.

### 3.3. Pathogen Movement within Palms and the Latent Period

At the beginning (t = 0), out of the six asymptomatic palms, five showed positive results when the bud leaf samples were used, while none of the other tissue types including inflorescence, root, and stem boring tissues showed positive results. However, after the one-year time period (t = 12 months), samples were taken from the same palms and tested. While the pathogen detection rate in bud leaf samples increased from 80% to 100%, some of the inflorescence and root tissues were also PCR-positive, as shown in Table 5. However, no positive samples were observed among stem borings.

One year later, all the bud leaf samples were positive for phytoplasma without expressing any characteristic visual symptoms of WCLWD. Furthermore, two palms showed positive results for inflorescence tissues and one palm showed a positive PCR for the root tissue after that period. Yet, all the palms remained apparently healthy and asymptomatic. In other words, all these palms were asymptomatic at least for a one-year period even though they were infected.

### 3.4. Screening for Other Fastidious Prokaryotes

No positive PCR results were obtained for any of the other targeted fastidious microorganisms. Continuous attempts with varying PCR conditions and annealing temperatures also failed in PCR-based detection for other fastidious prokaryotes.

## 4. Discussion

Rapid and reliable detection is the first and the most important step in disease management. Here, we present a suitable primer combination for nested PCR-based detection of phytoplasma causing WCLWD, a quarantined pathogen causing a severe threat to coconut production. Validation of a suitable primer pair was achieved and the P1/Tint [13] and fU5/rU3 [11] nested primers could identify the infection with 88% (*n* = 25 symptomatic palms) sensitivity. The other primer combinations tested did not yield positive results in PCR or in nested PCR. Therefore, no multiplexed PCR with primer combinations would even work. Although our intention was to achieve a 100% success rate, low pathogen titer, erratic distribution of the pathogen, and the large structure of palm trees may have challenged the detection and resulted in a negative PCR. In two independent experiments designed to infer the pathogen movement and determine the best tissue types, a minimum success rate of 75–88% in pathogen detection was achieved. However, in the case of white leaf disease affecting sugarcane plants, the method was always successful in detecting the pathogen (data not shown). Both white leaf disease-causing phytoplasma and Weligama coconut leaf wilt disease-causing phytoplasma showed 99–100% similarity in BLASTn searches, confirming the 100% specificity of the method. As there were no PCR-positive results obtained for other fastidious prokaryotes, an association of those microorganisms with WCLWD was not proven. However, it should be noted that we performed a direct PCR for those other fastidious prokaryotes as per the relevant literature. The failure of PCR is not an indication of the absence of other fastidious prokaryotes and there may still be a latent infection in low titers.

The rate of detection of phytoplasma was higher in young bud leaf tissues than in the other tissue types. Even though stem borings have been described as a feasible sample type for the detection of phytoplasma diseases in other palms [21,39], no positive results were obtained in the current study with coconut trees even with the palms that showed severe infection and symptoms. This might also be due to the low titer of the sampling site and uneven distribution of the pathogen in the stem. It was interesting to note that pathogen detection in the root and inflorescence samples was possible only when the bud leaf samples were positive. Therefore, ekels of milky white coconut bud leaf tissues were identified to be the best sampling tissue for population-scale molecular detection of WCLWD-causing phytoplasma. Unlike other phytoplasma diseases of palms, WCLWD shows slow disease progression and mild symptoms. However, the ultimate result is the reduction of yield and the death of the palm.

The 16S rRNA gene sequence analysis is the standard method used to classify prokaryotes phylogenetically [38,39,40]. According to the phytoplasma classification described by the phytoplasma taxonomy group of the IRPCM phytoplasma/spiroplasma working team in 2004, there was a separate group for rice yellow dwarf disease-causing phytoplasma. It was named the 16SrXI group and the *Candidatus* Phytoplasma oryzae species belonging to this group. The 16S rRNA sequences obtained from this study were clustered with the Ca. Phytoplasma oryzae sequence, and sugarcane white leaf disease-causing phytoplasma sequences belong to the 16SrXI group [30]. One of the coconut phytoplasmas causing coconut lethal yellowing belongs to group IV phytoplasmas and resolved the separation of the isolates in this study. Therefore, it was reconfirmed that the phytoplasma associated with WCLWD belongs to the 16SrXI group of phytoplasma. However, as suggested by Pilet et al. [39], multilocus species identification would give further insights into the evolutionary potential of the pathogen.

As all phytoplasma diseases are vector-transmitted and vectors transmit the pathogen while piercing the sieve elements of leaves, the pathogen will not be found in any tissue of coconut palms if there is no translocation through sieve tubes. However, according to the results obtained in this study, phytoplasma was observed in inflorescence and root tissues, as sieve tubes are interconnected with leaves and other organs of the palm, forming a network for the translocation of WCLWD phytoplasma, through which it can become systemic. This further helps to determine the latent period of the pathogen. The latent period of a disease is defined as the time period between the initial infection by the pathogen and the visual symptoms expression. According to the results, apparently healthy palms without WCLWD symptoms gave phytoplasma-positive results in PCR. Therefore, there may be an incubation period associated with WCLWD. This phenomenon has become a problem for field identification of affected palms. Furthermore, healthy-looking (apparently healthy) WCLWD-asymptomatic palms can harbor phytoplasma infection for longer periods without expressing any visual symptoms. During this period, the infection spreads to the inflorescence and roots of the palm. In this study, all the palms subjected to the test remained symptomless for at least one year. Due to the inability to predict the first date of infection, this time period may be longer than that. Continuous observation of these selected palms will provide information on the latent period up to the expression of symptoms. Therefore, the presence of an incubation (latent) period of at least one year or more was evident for WCLWD, being similar to other phytoplasma diseases in many countries. Having such a long latent period may be very harmful since it exposes infected but asymptomatic plants to pathogen acquisition by insects, resulting in an increased risk of disease transmission. The incubation period varies depending on the taxonomic group of infecting phytoplasma and its virulence. In lethal yellowing (LY) disease in Jamaica, it was observed that the incubation period was 114–191 days for young palms [19]. In Kerala root wilt (KRW) in India, the incubation period is 6–24 months or even 3 years in rare cases [20].

Finally, here, we reported a reliable and rapid pathogen detection system for WCLWD in coconut palms, and ekels of milky white bud leaf tissues were found to be the best sample type. Systemic movement of the pathogen was indirectly inferred by time scale sampling and the pathogen could live in the host without showing symptoms at least for a period of one year.

## Figures and Tables

**Table 1:** Nested PCR primer combinations used and selected annealing temperatures.

	Primers for the First PCR (Reference)	Primers for the Nested PCR (Reference)	Annealing Temperatures for Both PCR Cycles
1	P1/Tint [13]	fU5/rU3 [11]	56 °C for both cycles
2		R16F2n/R16r2 [10]	56 °C and 60 °C
3		P1694/Pc399 [15]	56 °C and 60 °C
4	P1/P7 [13]	fU5/rU3 [11]	50 °C and 56 °C
5		R16F2n/R16r2 [10]	50 °C and 60 °C
6		P1694/Pc399 [15]	50 °C and 60 °C

**Table 2:** Phytoplasma 16S rRNA sequences used in the phylogenetic analysis along with the host plants and relevant references.

Accession Number	Host Plant	Reference
GQ850122.1	Coconut	[24]
EU635503.1	Coconut	[7]
HF571986.2	Sugarcane	Unpublished
MT360451.1	Sugarcane	Unpublished
MT649298.1	Sugarcane	Unpublished
AF498307.1	Coconut	Unpublished
AF434989.1	Date palm	[25]
MG993138	Queen palm	[25]
MG993148	Cabbage palm	[25]
MG243700.1	Date palm	Unpublished
AF498309.1	Coconut	Unpublished
JX681021.1	Oil palm	Unpublished
KY814724.1	Coconut	[26]
MK617534.1	Coconut	[26]
MK992774.1	Coconut	Unpublished
JX273772.1	Coconut	[26]
KF993431.1	Coconut	Unpublished
EU328159.1	Coconut	[27]
EU636906.1	Coconut	[27]
KF419286.1	Coconut	Unpublished
JQ868442.1	Coconut	Unpublished
Y14175.1	Coconut	Unpublished
X80117.1	Coconut	[28]
GU952098.1	Coconut	Unpublished
LC228755.1	Coconut	Unpublished
KP064103.1	Coconut	Unpublished
DQ645636.1	Coconut	Unpublished
HQ613874.1	Coconut	Unpublished
D12581.2	Mulberry	[29]
MT862368.1	Sugarcane	[30]
MT860714.1	Sugarcane	[30]
MT860721.1	Sugarcane	[30]
CR1 (MZ822450)	Coconut	Current study
CR2 (MZ822449)	Coconut	Current study
CR3 (MZ822431)	Coconut	Current study
CR4 (MZ822432)	Coconut	Current study
CR5 (MZ822428)	Coconut	Current study
CR6 (MZ822430)	Coconut	Current study
CR7 (MZ822429)	Coconut	Current study
CR8 (OP279594)	Sugarcane	Current study
CR9 (OP279485)	Coconut	Current study
CR10 (OP279647)	Coconut	Current study
CR11 (OP279533)	Coconut	Current study

**Table 3:** Primers used in the study targeting other fastidious prokaryotes, their names, sequences, annealing temperatures, and references.

Primer Name	Sequence	Annealing Temperature	Target Organism	Reference
OI1	5′- GCGCGTATGCAATAC GAGCGGCA-3′	54 °C	*Liberibacter*	[34]
OI2c	5′-GCCTCGCGACTTCGCA ACCCAT-3′			
P89-f	5′ATTGACTCAACAAACGGGATAA3′	56 °C	*Spiroplasma*	[35]
P89-r	5′CGGCGTTTGTTTGTTAATTTTTGGTA3′			
Pfr1	5′-AATGGGTGTCGCTGCC ATT-3′	60 °C	*Phlomobacter*	[36]
Pfr4	5′-AGCAATGAAATTGTTA TTAACGC-3′			
Cxx1	5′ CCGAAGTGAGCAGAT TGACC 3′	57 °C	*Clavibacter*	[37]
Cxx2	5′ ACCCTGTGTTGTTTTC AACG 3′			
HL5	5′-AAGGCAATAAACGCG CACTA3′	60 °C	*Xylella*	[38]
HL6	5′-GGTTTTGCTGACTGGC AACA-3′			

**Table 4:** PCR positivity rate of different coconut tissue samples in triplicate for phytoplasma in WCLWD-affected and -free areas.

Sample Type	Tissue Type	Number of Samples	Number of Positive Samples	PCR Positivity Rate
WCLWD-symptomatic coconut palms	Bud leaves	12	9	75%
	Young inflorescence	12	3	25%
	Roots	12	5	41%
	Stem borings	12	0	0%
Asymptomatic coconut palms from a WCWLD-prevalent area	Bud leaves	12	9	75%
	Young inflorescence	12	2	16%
	Roots	12	5	41%
	Stem borings	12	0	0%
Healthy coconut palms from a WCLWD-free area	Bud leaves	6	0	0%
	Young inflorescence	6	0	0%
	Roots	6	0	0%
	Stem borings	6	0	0%

**Table 5:** PCR positivity or negativity rates obtained for each type of tissue taken from the WCLWD-asymptomatic palms in a one-year period.

Tissue Type	Number of Samples (at t = 0)	PCR+ Samples at t = 0 (%)	Number of Samples (at t = 12 Months)	PCR+ Samples (at t = 12 Months) (%)
Bud leaves	6	5 (83%)	6	6 (100%)
Inflorescence Stem borings	6 6	0 (0%) 0 (0%)	6 6	2 (33%) 0 (0%)
Roots	6	0 (0%)	6	1 (17%)

## Data Availability

All the data generated or analyzed during this study are included in this published article.
